# Deformation Properties of Concentrated Metal-in-Polymer Suspensions under Superimposed Compression and Shear

**DOI:** 10.3390/polym12051038

**Published:** 2020-05-02

**Authors:** Alexander Ya. Malkin, Valery G. Kulichikhin, Anton V. Mityukov, Sergey V. Kotomin

**Affiliations:** Institute of Petrochemical Synthesis, Russian Academy of Sciences, 119919 Moscow, Russia; klch@ips.ac.ru (V.G.K.); ant-mityukov@yandex.ru (A.V.M.); svk@ips.ac.ru (S.V.K.)

**Keywords:** concentrated suspensions, viscoelasticity, viscoplasticity, squeezing, shear stress, compression

## Abstract

Concentrated metal-in-polymer suspensions (55 vol.% and 60 vol.%) of aluminum powder dispersed in low molecular weight polyethylene glycol) demonstrate elastoplastic properties under compression and shear. The rheological behavior of concentrated suspensions was studied in a rotational rheometer with uniaxial compression (squeezing), as well as shearing superimposed on compression. At a high metal concentration, the elasticity of the material strongly increases under strain, compared with the plasticity. The elastic compression modulus increases with the growth of normal stress. Changes in the shear modulus depend on both normal and shear stresses. At a low compression force, the shear modulus is only slightly dependent on the shear stress. However, high compression stress leads to a decrease in the shear modulus by several orders with the growth of the shear stress. The decrease in the modulus seems to be rather unusual for compacted matter. This phenomenon could be explained by the rearrangement of the specific organization of the suspension under compression, leading to the creation of inhomogeneous structures and their displacement at flow, accompanied by wall slip. The obtained set of rheological characteristics of highly loaded metal-in-polymer suspensions is the basis for understanding the behavior of such systems in the powder injection molding process.

## 1. Introduction

Suspensions are permanent and important objects for rheological studies. A diverse range of suspensions can be seen in nature, applied to various fields for technical and everyday use, and as a subject of scientific interest for exploring the fundamental relationships between the structure and properties of the matter. This is a topic of numerous research papers and comprehensive reviews, covering various aspects of the rheology of non-Brownian suspensions, including rather concentrated compositions [1,2]. 

In our previous study, we considered the rheological properties of similar suspensions covering the whole concentration range in shearing [3].

The previous rheology analysis of concentrated suspensions showed concentrations close to 50–55% to be the intermediate range corresponding to the transition from materials which can flow (liquids) to materials which cannot flow (solids), though the latter can demonstrate irreversible deformations. The concentration range between 55% and 65% is especially important for several applications of concentrated suspensions because a manufacturer desires to have as high a concentration of a solid component as possible, while maintaining the material integrity and plasticity.

Here, it is worth mentioning that the term “plasticity” is frequently used in the rheological literature with two different meanings. The study of complex, multi-component media such as many colloid liquids, emulsions, suspensions, filled polymer melts, etc., has shown the existence of a transition from elastic deformation in a solid-like state to irreversible deformations of flow happening at some threshold of stress, which corresponds to the breakup of the rigid or soft material structure [4]. In this case, the term “plasticity” means the possibility of flow, i.e., the unlimited growth of deformations while the constant stress acts. However, another case is possible. A solid body is elastic, but at some levels of stress, part of its deformation becomes irreversible, yet restricted by the limited level dependent on stress. So, deformations exhibit limited growth at applied levels of stress (opposite to flow) and these ultimately irreversible (“plastic”) deformations correspond to the given stress. The second type of plasticity is observed in highly concentrated suspensions and only this definition of plasticity will be considered below.

There is an issue concerning the behavior of concentrated suspensions under various geometries of deformation. A traditional method of testing concentrated suspensions is uniaxial compression, where a sample is placed between two parallel plates and compressed so the material undergoes squeezing. The flow dynamics of a Newtonian liquid in this geometry, as well as for viscoplastic liquids, meet the classical von Mises condition of the solid-to-liquid transition, (e.g., as considered by Sherwood and Durban [5] and Adams et al. [6]). A similar approach was proposed by Roussel and Lanos [7]. Indeed, using the von Mises criterion is a rather standard approach when dealing with solid-to-plastic transitions. However, there are two limitations. First, this approach is related to linear elastic materials. Second, as mentioned above, the flow is usually meant to occur under plasticity. In this work, we are faced with a more general rheological case—the non-linear behavior of materials under study and limited irreversible deformations. There is increasing interest, not just in engineering materials, but also for multi-component suspensions that have various areas of application, including materials, compositions for the modern technology of power molding, etc.

The principal interest in formulating the basic equations and in analyzing the experimental results consists of understanding the conditions of the appearance of wall slip. General discussions concerning causes and consequences of wall slip were provided by Malkin and Patlazhan [8] and considered by Cloitre and Bonnecaze [9], and He at al. [10] directly related this to highly concentrated suspensions, which are the focus of the current study.

A special field for analyzing the rheology of moderately concentrated suspensions is high shear rates, which leads to an increase in the viscosity up to the loss of fluidity (jamming) or the jump-like mechanical glass transition (continuous or discontinuous shear thickening) [11,12,13,14,15,16,17,18,19]. However, when discussing these concentrated suspensions, it is assumed that a medium retains its integrity in all cases.

A rather interesting phenomenon observed in experiments with concentrated dispersions is the formation of separate clusters, which can be treated as relatively regular mesoscopic domains [20,21]. Such structure elements move relative to each other as a whole. In these conditions, the deformation becomes macro-inhomogeneous and localized and this results in shear banding, which has also been observed in the squeezing flow of entangled polymer melts [22]. Similarly, large-scale structure anisotropy in the deformation of concentrated gels has been observed [23]. The inevitable appearance of structure anisotropy in the deformation of granular media was proven by Alonso Marroquín et al. [24] and the heterogeneous deformation in concentrated suspensions was also described by Kawabata et al. [25]. Such heterogeneity of the deformations is evidently inherent for all multicomponent materials, including not only rigid but also soft matter, e.g., concentrated emulsions [26]. Computer modeling confirmed that structure anisotropy is the result of squeezing flow [27].

Inhomogeneity in the deformation of concentrated suspensions is analogous to the displacement of granular media [28,29]. Therefore, the following general conclusion concerning the mechanism of deformation of any multi-component materials should be accepted: ‘Shear localization is a generic feature of flows in yield stress fluids and soft glassy materials’ [30]. It is then necessary to agree with the next maxim: ‘A complex fluid exhibits unexpected heterogeneous flow’ [31]. This fact makes it difficult to study the rheological properties of concentrated suspensions but does not preclude the need for a quantitative study of the squeezing of highly concentrated suspensions.

The important component of the rheological properties of different fluids is the elasticity or elastic recoil, superimposed on their irreversible deformations. A lot of fundamental studies on the elasticity of polymer melts and solutions have been published from the early days of rheology. It is necessary to mention the classical works of Weissenberg, including the famous Weissenberg effect. In addition, later publications demonstrated elasticity in shear and elongation [32,33,34,35,36,37,38,39] and generalized it in the classical monograph of Lodge [40]. However, all these works belong to the field of polymer rheology and it is difficult to find studies on suspension elasticity. Meanwhile, elastic deformations are rather important for estimating the technological properties of concentrated suspension and that is why they will be considered within this study.

Thus, in studying the rheological behavior of concentrated non-colloidal suspensions, it is of interest to elucidate how squeezing affects the development of elastic and plastic shear deformations in concentrated suspensions. This work is an attempt to clear up these questions for the 55 and 60 vol.% concentrations of suspensions, which are of the general rheological interest, as was discussed above, as well as of great technological interest, in particular for the powder injection molding (PIM) of feedstocks. 

## 2. Materials and Methods

The main materials of this study were 55% and 60% suspensions of aluminum (Al) powder dispersed in the low molecular weight polyethylene glycol (PEG) with a viscosity of 0.11 Pa*s at 25 °C. The average size of Al particles was 24 mkm and its density was 2700 kg m^−3^. Suspensions were prepared by mechanically mixing Al powder with PEG. All operations were done manually due to the high viscosity of the mixture.

**Figure 1:**
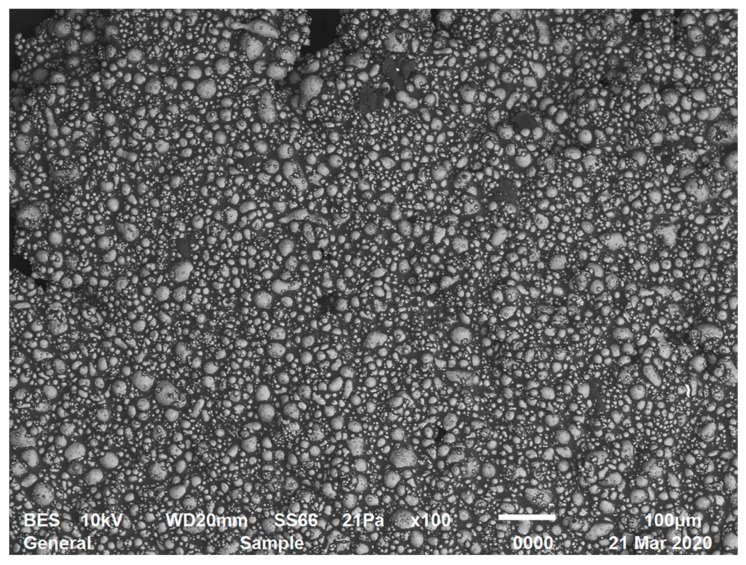
Microphotograph of a 60% suspension (scanning electron microscope JSM-6510 LV, JEOL, Japan).

A microphotograph of a sample of the typical 60% suspension is shown in Figure 1. Particle distribution is seen to be generally homogeneous.

All rheological experiments were performed on a RS-600 (Thermo Haake, Karlsruhe, Germany) rheometer at 25 ± 1 °C. Two versions of operating units were used: a plate–plate pair with smooth surfaces, with the radius R = 20 mm (the lower plate was made of steel and the disposable upper plate was made of Al) and a plate–plate pair with rough surfaces. The gap between plates was 2 mm and the area of contact was 1.26 × 10^−3^ m^2^.

The shear experiments were carried out at the given shear stress mode in the range of 1000–7000 Pa. Then, the development of shear deformation over time followed. The main task of these experiments was to clear up the role of compression on the rheological behavior of concentrated suspensions at shear.

The compression (squeezing) experiments were carried out with the same initial distance between plates. The measured value was the gap between plates after applying a normal force, which varied in the range corresponding to the normal stresses, σ_E_, from 4–25 kPa.

The stationary degree of compression was reached very fast and its value was a function of the applied stress. No further squeezing (flow) after reaching this stationary degree of compression took place. Then, the applied force was reduced to the minimal value of 8 Pa and the elastic recoil was measured. The difference between the initial gap and the gap after elastic recoil is a fraction of irreversible (plastic) deformation.

In addition to the rheological behavior under pure compression, we examined the behavior of compressed samples (maintaining a constant normal force) under shearing. The duration of shear loading was 120 s. The same time regime was used for elastic recoil in shearing. The latter allowed us to calculate the shear elastic modulus.

## 3. Results and Discussion

**Figure 2:**
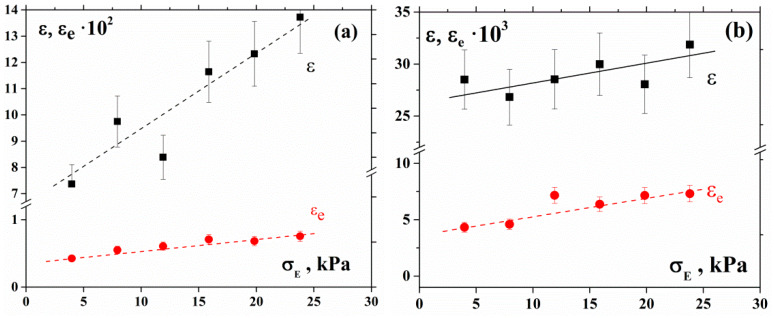
Total deformation ε (black marks) and its elastic part ε_e_ (red marks) observed under compression for 55% (**a**) and 60% (**b**) suspensions.

Let us consider the deformation under the compression of concentrated suspensions and changes in compression and shear modulus. The total deformation of the materials under compression ε, consisting of elastic $\varepsilon_{e}$ and plastic parts is shown in Figure 2.

**Figure 3:**
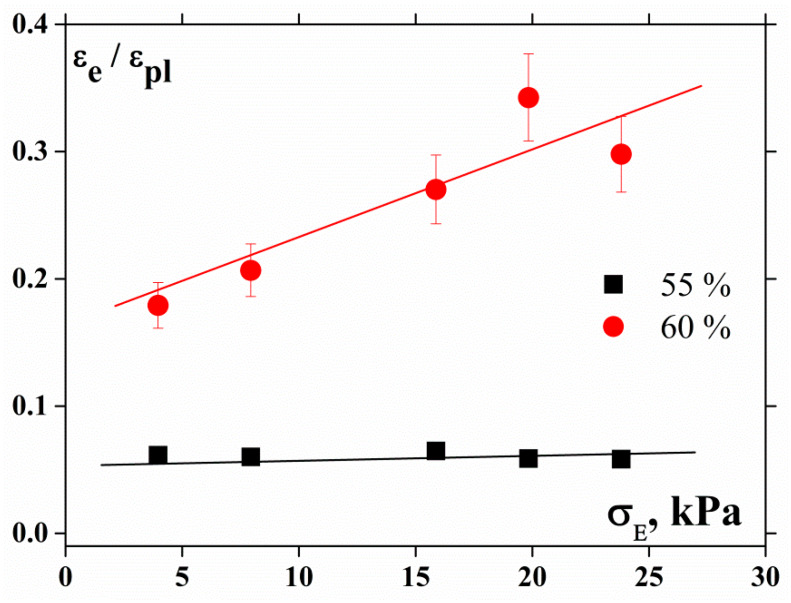
Relationship between elastic $\varepsilon_{e}$ and plastic $\varepsilon_{pl}$ components of the total deformation during compression.

The plastic deformation under compression is much higher than the elastic one, although the elasticity increases in the transition from 55% to 60% suspension, thus the plasticity is suppressed. This is evident from the analysis of Figure 3, where the dependence of the ratio of elastic to plastic strain under squeezing force is plotted, reflecting the transition from the viscoelastic to the elastoplastic rheological state. As shown in our previous paper [3], 50% is the threshold concentration for the transition from viscoplastic to elastoplastic behavior for the composition under study. 

**Figure 4:**
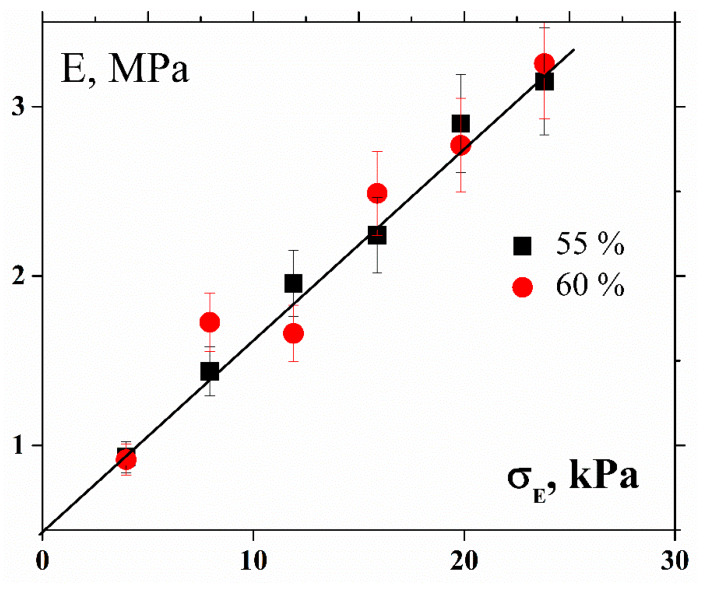
Elastic compression modulus vs. normal stress.

This transition is reflected in the value of the elastic modulus under compression (Figure 4). The non-linear behavior (changes in the modulus) of the material is supposed to be related to the influence of compression on the suspension structure. Indeed, under normal compression, the rigid particles get closer to each other and the polymer layers between them become thinner. Therefore, the material structure changes and this might lead to the rise of an effective compression modulus. 

**Figure 5:**
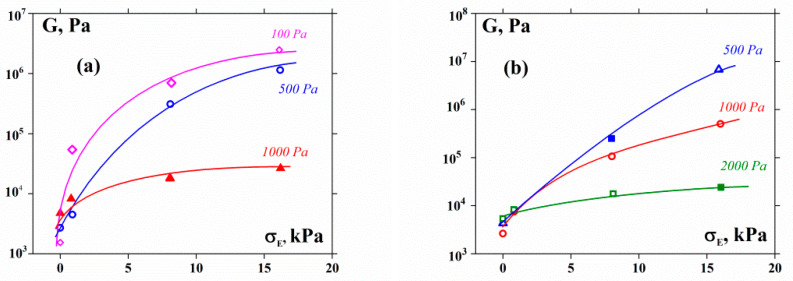
Shear modulus as a function of compression stress for 55% (**a**) and 60% (**b**) suspensions. Figures at the curves are the compression stresses.

In contrast, the behavior of the shear modulus under compression appears quite different. The results of measuring the shear modulus G for a sample that is simultaneously loaded by the normal force are shown in Figure 5, demonstrating the dependencies of the elastic modulus at different shear stresses $G\left(\sigma_{E}\right)$ on the compression stress, σ. As seen, the apparent shear modulus decreases with increasing normal stresses. 

It is worth noting that the real shear stress should be estimated while taking into account the action of normal force, which also contributes to the shear stress in the tangential direction. As known, the maximal value of the shear stress related to the normal stress is $\sigma_{\max}=\sigma_{E}/2$. The corrected total value of the shear modulus G_cor_ is

(1) $$G_{cor}=\frac{\sigma +\sigma_{E}/2}{\gamma_{e}}$$

where $\gamma_{e}$ is the elastic shear deformation.

**Figure 6:**
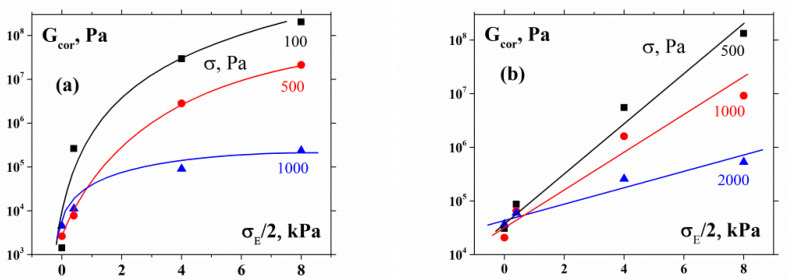
Corrected values of the shear modulus as a function of compression stress for 55% (**a**) and 60% (**b**) suspensions. Figures at the curves are the compression stresses.

The results of this correction are shown in Figure 6. The characteristics of the dependencies in Figure 5 and Figure 6 are similar.

One can see some differences in the behavior of the 55% and 60% suspensions. Indeed, the G values in the former case (to a certain degree) reach a plateau, while in the latter case, they continuously grow. This can be explained by the higher plasticity of the 55% suspensions, where limited possibilities for particle displacement remain. Close packing in the 60% suspensions creates a much more rigid structure, approaching the limit of the closest arrangement. Then suspension deformation can take place via the compression of the structure as a whole.

To compare the data obtained under shear and compression, let us consider the classical relationship between the Young’s modulus E and the shear modulus G for solid homogenous materials. The classical theory for solid polymers provides the following relationship [41]:

(2) E=2(1+μ)G

where μ is the Poisson coefficient, which cannot exceed 0.5 for polymers.

According to our experimental results, at the limit of low stress $E≅5*10^{5}Pa$ (Figure 4), while $G\approx 1*10^{3}Pa$ and $G\approx 4*10^{4}Pa$ for 55% and 60% suspensions, respectively (Figure 6). Therefore, linear Equation (2) does not work for both suspensions. It may, however, be more reasonable to suppose that the measured elastic modulus is actually close to the modulus of the volume (compression) deformations B. If one considers not the Young modulus, E, but the modulus of compressibility (or the modulus of hydrostatic compression, B), then the relationship between B and G in the linear theory of elasticity is expressed as [41]:

(3) $$B=\frac{2\left(1+\mu \right)}{3\left(1-2\mu \right)}G$$

If it is considered that μ≅0.45 for the 55% suspension, then the ratio B/G≅10 correlates well with the experimental data for this material. The value of the Poisson coefficient is even higher and closer to 0.5 for the more plastic 60% suspension, which explains the higher value of the B/G ratio for this object.

**Figure 7:**
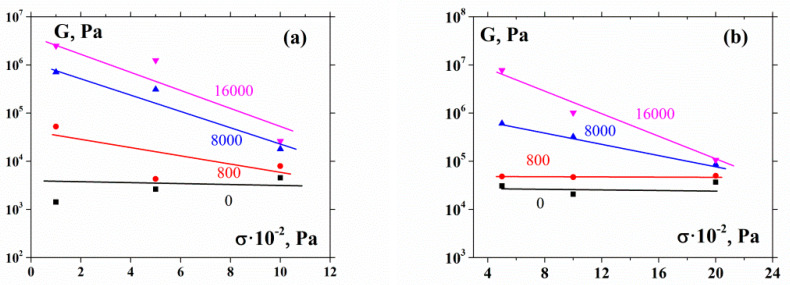
Shear modulus as function of shear stress at different compression stresses for 55% (**a**) and 60% (**b**) suspensions. Figures at the curves are the compression stresses.

A rather interesting effect of a sharp decrease (up to several decimal orders) in the shear modulus as a function of the shear stress at the pressure growth (Figure 7 reconstructed from Figure 6) has already been mentioned above. This does not happen at low compression, where the shear modulus only slightly depends on the shear stress. This effect is definitely related to the rearrangement of the spatial distribution of hard particles under compression. The phenomenon of the particle boundary mobility was considered by O’Brien and Foiles [42], who related its origin to temperature. The stress-driven mobility leading to the specific rearrangement of the space particle distribution should be a stronger factor.

The noticeable decrease in the shear modulus with shear stress growth (at a constant normal pressure) is a rather interesting phenomenon. To the best of our knowledge, this effect has not been described anywhere before. The decrease in the modulus seems quite unusual for dense suspensions. This effect can possibly be understood if it is assumed that the pressure leads to a heterogeneous mode of deformation, as was observed in numerous previous studies [28,29,30,31]. Thus, we may explain such a phenomenon by the rupture of weak cohesive bonds with the subsequent spurt of rather large blocks containing solid particles.

The experimental data show that the common peculiarity of the highly concentrated suspension is the superposition of elastic and plastic deformations. The latter is understood as irreversible (but is restricted in opposition to the flow) displacement under the imposition of constant stress. The restriction of deformations does not allow for following (irreversible deformations) and this is equivalent to local jamming. The latter is a well-known effect of highly concentrated suspensions [43,44,45].

The experimental data presented in this work, characterize the rheological behavior of highly concentrated suspensions. The general rheological equation for concentrated suspensions can be written as:

(4) $$D=D_{el}(\sigma_{E},\sigma )+D_{pl}(\sigma_{E},\sigma )$$

where *D* is the tensor of total deformation, *D_el_* and *D_pl_* are its elastic and plastic components, respectively, and both depend on the normal $\sigma_{E}$ and shear σ component of the stress tensor.

The form of these dependences depends on the concrete material.

## 4. Conclusions

Concentrated suspensions with solid phase contents of 55 and 60 vol.% are elastoplastic media that possess dominating plasticity. The latter is understood as the ability to undergo irreversible deformation under shearing and compression, which (in opposition to flow) is limited, and the value depends on the stress. Compression does not lead to squeezing flow, but does promote limited plastic deformations due to the local jamming. The shear modulus of compressed samples increases with the growth of normal stress. The comparison of shear and elastic moduli measured, respectively, in shearing and uniaxial compression showed that the elastic modulus is closer to the bulk modulus than the Young’s modulus.
